# Building Bridges: A framework for redefining and decolonizing implementation science in low- and middle-income countries

**DOI:** 10.1371/journal.pgph.0006155

**Published:** 2026-09-15

**Authors:** Binod Rayamajhee, Gopiram Syangtan, Bhupendra Lama, Saruna Ghimire, Brian Oldenburg, Uday Narayan Yadav

**Affiliations:** 1 Faculty of Medicine, Health and Human Sciences, Macquarie Medical School, Macquarie University, Sydney, Australia; 2 School of Optometry and Vision Science, Faculty of Medicine, University of New South Wales, Sydney, Australia; 3 Department of Infection and Immunology, Kathmandu Research Institute for Biological Sciences (KRIBS), Kathmandu, Nepal; 4 Department of Microbiology, Amrit Campus, Tribhuvan University, Kathmandu, Nepal; 5 National Institute of Bio-Science and Research Center (NIBARC), Kirtipur, Nepal; 6 Department of Sociology and Gerontology and Scripps Gerontology Center, Miami University, Oxford, Ohio, United States of America; 7 Department of Cardiovascular Research, Translation and Implementation, Baker Heart and Diabetes Institute, Melbourne, Victoria, Australia; 8 La Trobe University, Melbourne, Victoria, Australia; 9 National Centre for Epidemiology and Population Health, Australian National University (ANU), Canberra, Australia; 10 International Centre for Future Health Systems, University of New South Wales, Sydney, New South Wales, Australia; 11 Centre for Research, Policy and Implementation (CRPIN), Biratnagar, Nepal; University of Global Health Equity, RWANDA

## Abstract

Implementation science (IS) is gaining recognition for its role in enhancing health outcomes by connecting research evidence with practical application. However, most IS frameworks have been developed in high-income countries (HICs), raising concerns about their applicability, fairness, and sustainability in low- and middle-income countries (LMICs). In LMICs, which are home to over 75% of the global population, implementation challenges are influenced by unique social, cultural, geopolitical, and health system factors. This context reflects both opportunities and disparities in current IS models, such as dependence on external funding, lack of understanding and utilization of Indigenous Knowledge and Practice, and the underrepresentation of local knowledge holders/researchers as inventors. This critical review underscores the urgent need to decolonize and redefine IS in LMICs or Global South. We propose the Building Bridges framework encompassing advancing equitable and decolonized IS as foundational principle and four key pillars, including localizing and adapting IS frameworks, shifting power to LMICs leadership, equitable collaboration, capacity building, reforming funding agencies, and diversifying review panels, to foster a more just and locally relevant IS agenda. By prioritizing local leadership and valuing diverse knowledge systems, IS can reshape the local health landscape by embedding community-led decision-making and privileging locally grounded evidence based on lived experience, cultural practices, and relational worldviews, crucial for driving equitable, resilient, and sustainable health solutions in LMICs.

## Introduction

The world is facing a widening chasm in health and well-being, with LMICs bearing the greatest burden due to overstretched, under-resourced healthcare and social systems [1]. These systems often fail to meet the holistic needs of their populations, constrained by limited resources, inadequate infrastructure, and systemic challenges that span across patient-family, community, service provider, and broader healthcare system levels [2]. Despite the development of well-crafted policies and strategies that promote equity, integration, and people-centered care, the translation of these principles and evidence into implementation actions is limited [1]. This persistent gap between policies and their effective implementation, alongside limited resource-sharing to create context-sensitive solutions, significantly hinders progress toward improving health and well-being outcomes in LMICs.

Implementation science has emerged as an important field that bridges the gap between evidence and action [3] yet in LMICs, the implementation of policies and programs has historically been largely determined by high-income countries (HICs), with health agendas often being donor-driven and international agencies determining funding priorities and programmatic focus [4,5]. Consequently, the healthcare systems in many LMICs operate according to externally advocated agendas and objectives, limiting their ability to set and pursue locally determined priorities [6]. To achieve sustainable progress, power, decision-making authority, and resources must be shifted to LMICs institutions and leaders. This necessitates recognizing the value of local knowledge systems and empowering local actors to take the lead in IS initiatives. Rather than imposing externally deriving solutions, partnerships should prioritized Indigenous and other marginalized forms of expertise, enabling the co-design of culturally grounded implementation strategies that advance health equity [7]. Health research funding agencies in both HICs and LMICs are well-positioned to support this shift by advancing context-responsive research methodologies, investing in novel research gaps and ideas and facilitating the translation of evidence into practice. Global health initiatives like ESSENCE (Enhancing Support for Strengthening the Effectiveness of National Capacity Efforts) harmonize funders’ investment in health research capacity in LMICs, with a strong focus on promoting the uptake of evidence in policy and practice [8]. For example, the Global Alliance for Chronic Disease [9], which is a network of health research funding agencies and global IS researchers, has undergone a significant shift over the past decade in recognizing the importance of IS by strengthening the capacity of leaders and institutions in LMICs and increasing direct investment in LMICs-led research and priority-setting.

**Fig 1:**
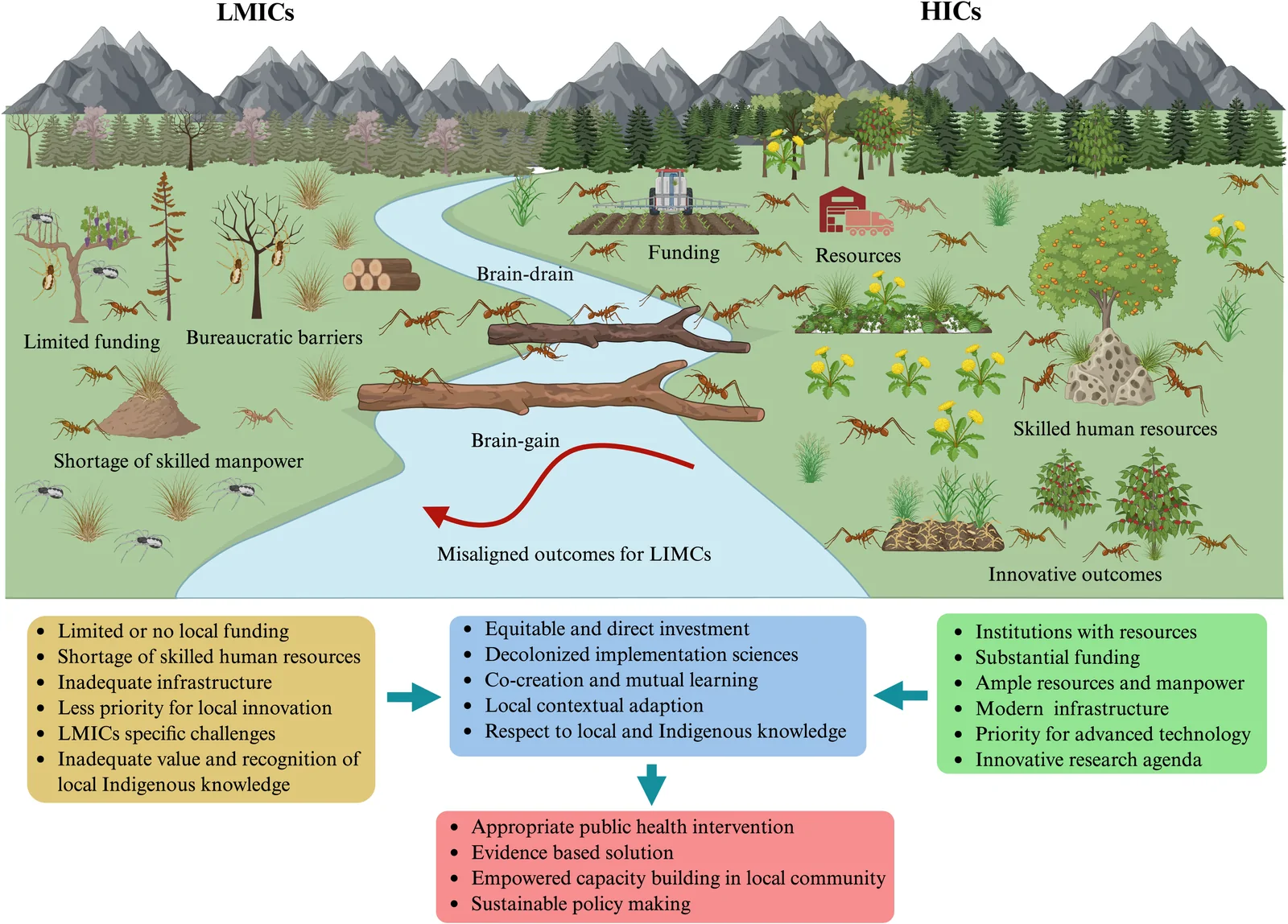
Illustration depicting disparities and interdependencies between LMICs and HICs in global health implementation science, using a biological ecosystem metaphor. The damaged bridge represents weak and unequal partnerships, resulting in misaligned outcomes for LMICs. The restored bridge illustrates a decolonized implementation science framework that promotes bidirectional knowledge exchange, equitable investment, mutual learning, and locally driven innovation, recognizing that implementation strategies and innovations developed in LMICs can also inform policy and practice in HICs. This reciprocal exchange supports sustainable, context-specific solutions and strengthens global health systems. Created in BioRender, Rayamajhee, B. (2026) https://BioRender.com/lprfgvs.

This review applied a concept-driven critical review that typically results in the development of a framework that synthesizes emerging critiques of IS as it is currently practiced in LMICs, examines innovative decolonial adaptations in South Asia, Latin America, and sub-Saharan Africa [10]. The search was conducted across Google Scholar, PubMed, Web of Science and Scopus. Based on critical review of literature, we proposes the “Building Bridges” framework (Fig 1), developed iteratively and informed by the conceptual framework development approach described by Jabareen (2009) [11]. Specifically, concepts relevant to decolonizing implementation science in LMICs were identified and mapped from the literature reviewed, conceptually related ideas were grouped into thematic domains using a conventional content analysis approach, and the relationships and boundaries between these domains were iteratively examined and refined [12]. The resulting domains were then critically reviewed and refined through consensus discussions among the author team, drawing additionally on the authors’ collective experience in IS and health research in LMICs settings. The primary purpose of this critical review is to critically synthesise the literature on decolonizing IS and advance the discourse by providing scholars, practitioners, funders, and policymakers with a practical, principled roadmap for more equitable, contextually responsive, and sustainable implementation efforts in LMICs. By bridging divides between Northern theories and Southern realities, this work aims to catalyze systemic change toward epistemic justice, health equity, and truly global health progress. Ultimately, this review is organized to foster a paradigm shift toward locally driven, community-embedded implementation practices that bridge the persistent evidence-to-action gap, advance health equity, strengthen resilient health systems, and contribute meaningfully to global health goals such as universal health coverage and the United Nations Sustainable Development Goals.

## Critiques of current implementation science in LMICs

LMICs have a rich history of designing and evaluating public health programs, including task shifting and community-centered interventions, leading pivotal implementation trials on infectious and non-communicable disease prevention and control that have long been the operational backbone of global health interventions. For example, the Prevention of Mother-to-Child Transmission (PMTCT) studies tested in many LMICs demonstrated the feasibility of PMTCT programs to be scaled up at the population level [13]. However, the structural foundations of IS have historically failed to support or reflect this reality. Power has remained concentrated in institutions based in HICs, where researchers set research priorities, design methodology, authorship hierarchies, and evaluation metrics, leaving LMICs leadership and expertise under-recognized and under-resourced [14,15]. Additionally, IS frameworks were almost exclusively developed in Western academic contexts, leading to theoretical models that were insufficiently adapted to the sociopolitical and other contextual realities of LMICs, including Indigenous knowledge systems, community governance structures, or health system constraints. Similarly, collaboration models often reproduced extractive dynamics such as LMICs actors provided data, led fieldwork, and offered contextual insights, yet meaningful two-way knowledge exchange initiatives, such as on scientific writing, evidence-based practice, and scientific research methodology training, and decision-making authority were rarely shared [16,17]. Furthermore, funding mechanisms and peer review processes reinforced these imbalances; grant agencies in the Global North favored Northern principal investigators, prioritized Western-centric research questions, and convened review panels lacking LMICs representation, which limited contextually grounded innovation and discouraged locally led proposals [15]. Consequently, these systemic barriers have prevented the emergence of a truly equitable, decolonized IS ecosystem as the global knowledge system surrounding IS has consistently failed to acknowledge, incorporate, or elevated LMICs leadership, frameworks and epistemologies [18].

Although considerable progress has been made, there is still a long way to go in building IS capacity in LMICs and measuring the impact of locally driven practices. Moreover, many funders have yet to fully recognize the value of prioritizing implementation research, often due to limited internal expertise and unclear principles to guide robust investment in global health IR, an essential step for generating context-specific evidence to inform effective policy and practice [8]. While debates continue about the definition and boundaries of decolonizing IS, a growing consensus is emerging on the need for a decolonial approach to the implementation, scaling up, and sustainability of global health research. For instance, although the World Health Organization’s ‘best buys’ for reducing non-communicable diseases suggest several interventions, the lack of context-specific evidence for effective translation often limits their adaptation and implementation in LMICs [19].

It is important to emphasize that implementation science conducted in the Global South by local actors is not inherently decolonized [20]. Rather, decolonized IS is defined by the locus of power, epistemic justice, and structural equity. It constitutes research in which LMICs actors set the agenda, own the data, define the implementation outcomes, and validate local knowledge systems [21,22]. By contrast, non-decolonized IS may still arise within the Global South when local researchers uncritically adopt Western-flavored Theories, Models, and frameworks without meaningful cultural adaptation, or when institutions prioritize funder-driven metrics and publication in high-income-country journals over local policy uptake, thereby reproducing colonial dynamics from within [20,23].

## Case studies of successful decolonized IS initiatives in LMICs

Evidence shows that LMICs have been successful in designing and implementing various health and social innovations (e.g., technologies, programs, methodologies, and strategies) to address similar health and health-related challenges in HICs. For example, the Community Health Workers program originated in China in the 1920s to address various public health issues was was subsequently adopted across LMICs, including Nepal, Indonesia, Bangladesh, and Latin America, and is now increasingly implemented and recognised in HICs such as the United States, Canada, and Australia [24,25]. Recently, during the COVID-19 pandemic, several LMICs-led strategies, such as mother care for reducing morbidity and mortality, and the use of Oral Rehydration Solution (ORS) [26] were rapidly adopted by HICs, which embraced community engagement practices, rapid public health messaging approaches, and contact tracing methods from LMICs to yield impressive outcomes [25].

Despite the dominance of Western IS frameworks, LMICs have long generated innovative, context-driven models that demonstrate how locally led strategies can achieve sustainable health impact, and Global South researchers also serve as vital bridges between academic institutions, contributing irreplaceable contextual knowledge while building collaborative networks [27]. To illustrate the practical application of decolonized IS, several case studies from diverse LMICs demonstrate how locally led approaches can yield sustainable outcomes. For example, Nepal’s Female Community Health Volunteer (FCHV) programme, established in 1988, illustrates how community-rooted, low-cost interventions can drive major gains in maternal, neonatal, and child health while advancing progress toward universal health coverage (UHC) [28,29]. Similar strengths emerge from community-based HIV programmes in sub-Saharan Africa, where integrating community health workers and local leaders into ART delivery systems has significantly improved retention and viral suppression, underscoring the effectiveness of culturally grounded implementation models [30]. In Latin America, homegrown policy implementation frameworks addressing urban poverty, food insecurity, and multisectoral coordination have enabled successful non-communicable disease (NCD) interventions and informed region-wide strategies that reduced NCD mortality and strengthened primary care [31]. South Asia offers additional evidence of LMIC leadership such as International Center for Diarrheal Disease Research, Bangladesh (icddr,b) in Bangladesh has pioneered cost-effective, scalable public health solutions from oral rehydration therapy to community-based NCD management by embedding cutting-edge research and policy engagement within national systems [32]. In India, the Indian Council of Medical Research (ICMR) and All India Institute of Medical Sciences (AIIMS) have advanced implementation science through frugal innovation, digital health platforms, and large-scale programs such as Ayushman Bharat, demonstrating that decentralized, equity-focused health system models can deliver high-quality care even with limited resources [33]. In Kenya, the African Population and Health Research Center (APHRC) have pioneered community-engaged IS for urban health challenges, integrating local slum dwellers’ knowledge into sanitation and nutrition interventions. This model shifted power by training community members as co-researchers, resulting in a 30% reduction in waterborne diseases through context-adapted hygiene programs-outcomes that traditional top-down frameworks might have overlooked [34].

In Brazil, the Oswaldo Cruz Foundation (Fiocruz) has decolonized IS in public health by integrating Indigenous perspectives into vector-borne disease control, including Zika and dengue. Through collaboration with Amazonian communities, Fiocruz adapted surveillance frameworks to incorporate traditional ecological knowledge, yielding more effective, low-cost mosquito control strategies that reduced incidence by 25% in pilot areas. This case underscores the importance of equitable partnerships, in which LMICs institutions lead in methodology design and data ownership [35,36]. Another example from Vietnam involves the implementation of mental health services post-COVID-19, where the Hanoi School of Public Health localized Western IS tools by blending them with Buddhist-influenced community support systems. This hybrid approach enhanced stigma reduction and service uptake, highlighting how cultural adaptation can improve the scalability of services. These cases collectively show that decolonized IS not only addresses local disparities but also generates transferable lessons for global health [37]. Collectively, these local examples highlight that LMICs possess rich, contextually grounded expertise that should inform global implementation science theory and practice but remains undervalued in dominant IS frameworks.

## Power, partnership, and progress for leadership: Shifting the lens for LMICs

Funding agencies have begun reforming their structures to better support researchers from LMICs who possess the contextual knowledge and subject expertise required to navigate local implementation dynamics [9].. However, much more is needed to achieve meaningful ‘implementation of IS’ that promotes equitable participation in global science and technology networks through equitable funding allocation, technology transfer, skills development, locally driven research agendas, and the active involvement of LMICs researchers in international partnerships for recognized leadership roles [38]. Decolonizing IS requires more than refining existing frameworks; it calls for a fundamental shift in how IS is conceptualized and applied within the LMICs context. Theoretical models, determinant frameworks, and evidence-based interventions must be culturally validated and contextually adapted to address the sociocultural, environmental, and structural dimensions of health systems [38,39]. To guide this sustainable transformation, we propose a five-domain framework for decolonizing IS in LMICs (Table 1), aimed at redefining and advancing health equity, strengthening research capacity, and fostering more inclusive science and policy partnerships.

**Table 1:** A framework for redefining and decolonizing IS in LMICs.

Key pillars	Actions
**Foundation:** Advancing equitable and decolonized IS in LMICs	IS must be decolonized to build sustainable local ecosystems that can shape future health systems, policy, and service delivery. The following key principles are critical to achieving this goal: i. Promote research rigor that integrates local knowledge, Indigenous healing practices, and allows flexible adaptation of interventions based on evolving local contexts. Adopt a people-centered, multicentric approach to implementing new technologies and systems, ensuring accessibility and cultural relevance. ii. Align implementation efforts with community priorities, health system needs, and policymaker goals to maximize real-world impact. iii. Promote dissemination of research findings in local languages and accessible formats to strengthen community ownership, accountability, knowledge translation, and the uptake of evidence into policy and practice. iv. Extend evaluation beyond traditional implementation and health outcomes to include measurable indicators of power redistribution, such as the proportion of implementation decisions led by local stakeholders, representation of LMICs researchers and community members in governance and leadership roles, equitable authorship and funding allocation, meaningful engagement of interest holders through participatory approaches, demonstrable community benefits, and policy or practice changes arising from locally generated evidence [40]. v. Prioritize epistemic justice by formally recognising multiple ways of knowing and evaluating the extent to which traditional, Indigenous, and community knowledge is integrated into research priority setting, intervention co-design, implementation, interpretation of findings, and policy development alongside biomedical evidence [41]. **vi.** Ensure cost efficiency while prioritizing holistic health and well-being. This foundation underpins all IS activities ensuring that epistemic justice and power redistribution and embedded in every phase of implementation, from context assessment to sustainability.
**Pillar 1:** Localize and adapt IS frameworks	i. IS frameworks should be contextualized in collaboration with local stakeholders, including communities, frontline workers, traditional healers, and policymakers. ii. Incorporate local socio-cultural, political, and systemic realities in research design, delivery, and evaluation. iii. Recognize Indigenous knowledge systems and community perspectives as valuable sources of insight, rather than viewing them as barriers to scientific advancement. iv. Ensure implementation strategies are deeply informed by local histories, belief systems, and lived experiences. v. Culturally adapt interventions to local contexts.
**Pillar 2:** Shift power to LMICs’ leadership	i. International funding agencies should invest in building the capacity of researchers and LMIC-based institutions, with the provision of enabling them to co-lead or lead global health projects with decision-making authority, grounded in local context and system knowledge. ii. National governments and academic institutions should invest in developing a robust local research workforce, equipping emerging leaders with skills in research design, implementation, and strategic decision-making. iii. LMICs leadership must be actively included in knowledge translation activities at local, regional, and international levels.
**Pillar 3:** Equitable collaboration and capacity building	i. Strengthen regional and international research networks that support LMIC-based researchers through funding, infrastructure, and technical assistance. ii. Implement mentorship programs and transformational leadership development to empower LMIC_S_ scholars and practitioners. iii. Promote bi-directional knowledge exchange between HICs and LMICs, ensuring reciprocal learning and respect for diverse knowledge systems. iv. Capacity building should extend beyond technical training to encompass leadership development, institutional strengthening, and long-term sustainability.
**Pillar 4:** Reform funding agencies and diversify review panels	i. Funding bodies and academic journals must adopt inclusive and ethical practices by ensuring LMICs’ representation in grant review panels, editorial boards, and decision-making processes. ii. Research protocols should be adapted to align with locally available resources, ensuring that research efforts lead to meaningful social impact by promoting equity, autonomy, local data ownership, and sustainable outcomes within the communities they aim to serve. iii. Funding criteria should value integration of IS research into existing health and social systems, social impact, sustainability, and alignment with local priorities, not solely scientific innovation. iv. Ensure review panels reflect epistemic diversity and value pluralistic knowledge systems, including non-Western approaches to health.

## Fostering equitable collaboration in implementation science

Equitable collaboration is recognized as a pillar in contemporary efforts to decolonize implementation science in LMICs. Historically, global health researchers have operated through extractive practices in which LMICs settings primarily serve as data collection sites, while research priorities, funding allocation, and knowledge production remain concentrated in HICs [42]. Efforts to decolonize implementation science and global health have brought renewed attention to structured collaboration, emphasizing questions of power and epistemic authority. Equitable collaboration is both a normative ethical imperative and a practical condition for generating impactful and sustainable implementation outcomes in LMICs [43–46].

Despite increasing recognition of equity as a pillar, leadership, agenda setting, and framework development remain concentrated in HICs. This imbalance constrains the relevance of research to local policy and practice and undermines the potential for LMICs-led innovations [47]. Addressing these limitations LMICs requires diversification of leadership, including funding and institutional investment, agenda setting, development, adaptation, and validation of context-specific frameworks that reflect local health systems, sociopolitical realities, and community knowledge. Equity must be positioned as a core outcome of implementation research rather than as an ancillary consideration [7,45]. Although calls for decolonization have gained momentum, structural inequities persist [45].

Research on global health partnerships consistently identifies both relational and structural determinants of equitable collaboration. Relational determinant includes trust, mutual respect, and a shared sense of belonging; structural determinants encompass formal governance arrangements, including clear contracts, capacity building, power sharing, inclusive divisions of labor, inclusive decision-making processes, and sustainable investment [44]. Donor-driven agenda setting marginalizes local priorities, while exclusionary labor practices limit career advancement opportunities for LMIC researchers. Inequitable authorship, weak accountability mechanisms, and chronic underfunding of indirect costs for LMICs institutions further exacerbate institutional vulnerabilities and dependency [41,44].

In response, various normative and practical tools, such as the Douala Equity Checklist for international partnerships and structured equity frameworks for adaptive platform trials, propose concrete criteria related to funding, leadership, ethics, authorship, data access, and capacity strengthening [41,46]. In a parallel participatory implementation science approach, including co-design and co-creation integrate perspectives of communities, implementers, and policymakers across the research cycle. These approaches move beyond “making evidence-based interventions fit” toward transforming systems to center equity and justice [40,48,49]. Challenges of these approaches include resource constraints, rigid hierarchies, and difficulties in accounting for structural determinants such as poverty, political interference, and historical marginalization, which complicate efforts to implement inclusive processes [40,48].

Colonial power relations are reproduced continuously through epistemic hierarchies, infrastructure control, and knowledge hierarchies [50], dominance of select expert gatekeepers and elite committees in approving and disseminating information, global governance structure, cultural and linguistic dominance, and data control [51,52]. Addressing these dynamics requires coordinated reforms across funding, curricula, dissemination, and governance to reconfigure global health futures in more just directions. While the mentorship, capacity building, and collaborative networks explicitly designed to shift power are increasingly advocated, substantial gaps remain between decolonization aspirations and the depth of structural transformation achieved in practice [45,52].

## Implications for the field and recommendations

Geopolitical factors also play a key role, particularly in conflict-affected or politically unstable LMICs, where implementation efforts are disrupted by external influences like sanctions or aid conditionalities [53]. For example, in regions such as sub-Saharan Africa, colonial legacies manifest in health systems that favor urban, elite populations over rural or marginalized communities, complicating the adaptation of IS frameworks [54]. Moreover, low-income populations, especially those living in climate-vulnerable regions such as sub-Saharan Africa and South Asian developing countries, such as Nepal, experience heightened risks from extreme weather events, food insecurity, and water-related diseases, and a high burden of non-communicable disease (for example, NCDs accounting for 66% to over 70% of total deaths in Nepal) which further challenge the effective implication of IS at the ground level [55]. Future research would benefit from fostering local ownership during the pre-implementation phase, thereby enabling context-specific, locally driven, and sustainable health system responses to climate-related and pandemic challenges [55]. Overcoming these barriers requires intentional efforts to dismantle extractive research practices, such as “helicopter research” in which teams from HICs collect data in LMICs without meaningful local involvement or benefit-sharing [56]. Limited leadership by LMIC researchers and institutions perpetuates extractive research practices, constrains local innovation, and reinforces dependency on external expertise [57]. These dynamics also have implications for capacity strengthening, which often prioritizes individual technical skills over institutional and system-level development [58]. The proposed Building Bridges framework reframes IS as a relational and reflexive field, one that bridges global evidence with local knowledge, formal research with community expertise, and scientific rigor with social accountability, especially in resource-scarce settings [59]. By adopting a decolonial lens, IS can evolve from a model of knowledge transfer to one of co-creation and mutual learning, supporting more adaptive, inclusive, and sustainable health system transformations in LMICs [7,51].

To advance a decolonized and context-responsive IS agenda in LMICs, coordinated action is required beyond sectors and across research practice, funding mechanisms, and institutional norms. First, local leadership should be centered throughout the research ecosystem, including priority setting, study design, data interpretation, and dissemination [7]. The global funding institutions must move beyond tokenistic inclusion by actively supporting research led by LMICs and ensuring that decision-making authority is genuinely vested in local stakeholders with ownership [14]. Second, IS should adopt epistemic pluralism by acknowledging Indigenous and cultural values, community-based knowledge, and lived experience as valid and essential forms of evidence alongside standard scientific approaches [60]. Most importantly, co-design and participatory methodologies should be normalized rather than treated as a complementary part of the process. Third, existing implementation frameworks should be critically evaluated and adapted or newly developed through locally grounded, participatory processes that explicitly account for contextual factors, cultural dynamics, and historical influences [42,61]. The “building bridge” framework presented in this paper provides a practical approach to navigating these complexities by fostering dialogue between global and local knowledge systems. On the other hand, South-based research partnerships should be reconfigured to address structural power imbalances through equitable distribution of resources, transparent authorship practices, and shared ownership of research outcomes. Finally, capacity-strengthening efforts should extend beyond individual training to encompass institutional research governance, mentorship networks, and mechanisms for policy engagement within LMICs settings [43].

While global health primarily concerns what health issues are addressed, IS focuses on how interventions are implemented, scaled, and sustained. Consequently, decolonizing IS necessitates specific structural shifts in the measurement of implementation determinants, the selection of implementation strategies, and the careful balancing of fidelity with local adaptation. The proposed Building Bridges framework reconceptualizes IS as a relational and reflexive field that bridges global evidence with local knowledge, formal research with community expertise, and scientific rigor with social accountability, particularly in resource-limited settings [59].

Coloniality in IS is deeply reinforced by structural dependency [47,62]. Current funding mechanisms frequently require HIC institutions to serve as prime awardees, thereby relegating low- and LMICs institutions to subcontractor roles. Short-term grant cycles further hinder the evaluation of long-term implementation sustainability [15,63]. To decolonize IS in a meaningful way, funders must shift toward direct, unrestricted funding for LMICs institutions, enabling them to develop sustainable implementation research infrastructures rather than remaining dependent on project-to-project survival [15,63,64]. The Building Bridges framework (Fig 1) visually captures the paradigm shift required to overcome this dependency. The damaged bridge symbolizes the prevailing extractive IS ecosystem, in which unidirectional knowledge transfer produces misaligned outcomes [47,62]. In contrast, the restored bridge represents the proposed framework, in which a foundation of equitable IS supports bidirectional knowledge exchange [47]. This visualization emphasizes that implementation strategies and innovations generated in LMICs must be recognized as valuable evidence capable of informing IS policy and practice in HICs, thereby fostering reciprocal learning and the development of sustainable, context-specific solutions [47,62]. Finally, decolonizing IS demands a sustained commitment to positional reflexivity. Practitioners in both HICs and LMICs must critically examine their own positionality, power, and the epistemic assumptions they bring to the implementation process [65,66]. Reflexivity should be embedded as a core methodological practice to ensure that researchers do not inadvertently reproduce the hierarchies they aim to dismantle [62,64]. By adopting a decolonial lens, IS can shift from a model of unidirectional knowledge transfer to one of co-creation and mutual learning, thereby supporting more adaptive, inclusive, and sustainable health system transformations in LMICs [7,51].

## Conclusions

LMICs’ leadership is progressing well towards addressing unmet health and well-being needs of their people through strengthening both community and health system approaches. However, substantial work is still required to achieve the UN Sustainable Development Goals. The proposed framework is grounded in the foundation of advancing equitable and decolonized IS and supported by four key pillars presents an opportunity for funding agencies, academics, policymakers, and other stakeholders to apply a decolonizing IS framework to support the rapid adaptation, effective implementation, and scale-up of innovations, while fostering open, transparent, and accountable relationships among researchers, policymakers, and implementers in LMICs.

## Abbreviations

AIIMS: All India Institute of Medical Sciences
ESSENCE: Enhancing Support for Strengthening the Effectiveness of National Capacity Efforts
FCHV: Female community health volunteer
HIC: High-income countries
ICDDR, B: International Center for Diarrheal Disease Research, Bangladesh
ICMR: Indian Council of Medical Research
IS: Implementation science
LMIC: Low- and middle-income countries
NCD: Non-communicable disease
ORS: Oral rehydration solution
UHC: Universal health coverage
UN: United Nations
